# Does Fiscal Stress Improve the Environmental Efficiency? Perspective Based on the Urban Horizontal Fiscal Imbalance

**DOI:** 10.3390/ijerph19106268

**Published:** 2022-05-21

**Authors:** Youshuai Sun, Demi Zhu, Zhenyu Zhang, Na Yan

**Affiliations:** 1 School of Economics and Management, Tongji University, Shanghai 200092, China; sunyoushuai@tongji.edu.cn; 2 School of International and Public Affairs, Shanghai Jiao Tong University, Shanghai 200030, China; zhudemi@sjtu.edu.cn; 3 School of Automation, Nanjing University of Science and Technology, Nanjing 210094, China; zhangzhenyu@njust.edu.cn

**Keywords:** environmental efficiency, fiscal stress, environmental governance, horizontal fiscal imbalance, data envelopment analysis method (DEA)

## Abstract

The resilience of the fiscal system has a driving effect on environmental governance, and it is always a challenge to solve the problem of matching fiscal power with administrative power. Based on the panel data of 193 cities in China from 2013 to 2018, the data envelopment analysis method was used to evaluate the comprehensive indicators of urban environmental efficiency. The impact of fiscal stress on environmental efficiency is examined from the perspective of urban horizontal imbalance. We find that the smaller the fiscal stress, the higher urban environmental efficiency. The endogeneity is mitigated by using instrumental variables and the generalized method of moments, and the results are still robust after considering the interference of sample selection bias and variable estimation bias. At the same time, the impact of fiscal stress on environmental efficiency varies with spatial location, ecological strategic planning, economic development, and other factors, especially in southern cities, cities in the Yangtze River Basin, and cities in urban agglomerations, where reducing fiscal stress promotes environmental efficiency. In addition, green production and public environmental services are important channels for its role, and the rational allocation of self-raised funds can effectively moderate the improvement of environmental efficiency.

## 1. Introduction

Global warming is one of the major crises facing humanity today. Since 1880, greenhouse gas emissions have caused the Earth’s temperature to rise by 1.4 degrees Celsius, which poses a continuing threat to global health and human life. In response to global warming, the 2015 Paris Climate Agreement required all signatories to reduce global temperatures below 2 °C [1]. To meet this goal, the Chinese government has made great efforts and devoted resources to energy conservation, emission reduction, and environmental protection. At the same time, the awareness of energy conservation and environmental protection among Chinese citizens has increased year by year. However, as the world’s second-largest economy, China’s energy consumption and greenhouse gas emissions remain challenges to the process of economic and environmental sustainability [2]. China has been the fastest-growing country in the world for 18 consecutive years in terms of energy consumption [3]. Since the 1980s, China’s rapid urbanization and industrialization have posed a serious threat to the fragile urban environment. At the same time, these processes have led to multiangle environmental pollution problems such as mineral energy depletion, excessive deforestation of forest resources, haze pollution, and water pollution, which have also seriously affected the Chinese public health and high-quality economic development [4]. In order to achieve the dual goals of economic development and environmental protection, environmental efficiency has become a key indicator for quantifying environmental governance [5].

The theoretical origins of environmental efficiency come from an extension of the concept of green growth. In the “Made in China 2025” strategic plan, the Chinese government has clearly proposed the concept of green growth. Green growth needs to eliminate dependence on an extensive development model with high energy consumption and low efficiency [6]. Generally, green total factor productivity (GTFP) is one of the important indicators to quantify green growth. However, whether GTFP can fully reflect the performance of regional environmental governance is a question worth pondering. In other words, GTFP pays more attention to green and clean production capacity in industrial enterprises than to the urban overall public environmental service capacity. As a public product, the ability of public environmental services can be reflected in specific links, such as sewage disposal and domestic garbage disposal. Comprehensive environmental efficiency indicators need to take into account the level of green and clean production and public environmental service capabilities. Therefore, GTFP is used to quantify the urban green production capacity. Public environmental service efficiency (PESE) is used to quantify urban public environmental service capacity. These two indicators represent two important paths to improving urban environment efficiency. It is in line with the route design of sustainable development of Chinese cities and helps promote the regional economy to achieve high-quality development goals.

The government is the main body in regional environmental governance and plays an important role. In the fiscal federalism literature, fiscal policy has become one of the important tools of government environmental management [7]. In the Chinese context, environmental efficiency is affected by the arrangement of the fiscal system. The core of the fiscal system arrangement lies in the scale of fiscal revenue and fiscal expenditure, which determine the fiscal stress of a region. Fiscal stress refers to the fiscal gap formed by the continuous imbalance between the local government’s fiscal revenue and fiscal expenditure or the mismatch between fiscal revenue and expenditure. According to China’s “Budget Law”, China’s public fiscal budget is set at five levels: the central government, provinces, prefecture-level cities, counties, and townships. The fiscal budget at the city level refers to the composition of the prefecture-level city itself and its subordinate counties. Since 1994, the Chinese central government has regularly initiated various fiscal reforms to adapt to the changing state of the economy. In 2003, the ratio of corporate income tax of the central government and local governments was adjusted to 60:40. Since the 2016 tax reform of replacing business tax with value-added tax, the ratio of value-added tax between the central government and local governments has been 50:50. Whether it is the reform of the sharing of corporate income tax or the reform of replacing business tax with value-added tax, the actual tax revenue of local governments has been greatly reduced, while local fiscal expenditures have continued to rise, which has greatly increased the fiscal stress of local governments. Environmental efficiency measures the government’s public governance capacity, and fiscal capacity is the basis for the government to solve environmental governance problems. Facing the dual challenges of economic structural transformation and the COVID-19 epidemic and whether fiscal policy can improve the environmental efficiency of Chinese cities is a research topic that needs to be explored urgently.

A large array of literature demonstrates the impact of fiscal decentralization on environmental efficiency from various perspectives [8,9,10]. However, the impact of fiscal stress on environmental efficiency has not been fully discussed [11], and empirical research in related areas is scarce. We use panel data from 193 cities in China from 2013 to 2018 and evaluate the comprehensive index of urban environmental efficiency using the data envelopment analysis method (DEA). The impact of fiscal stress on environmental efficiency is examined. We found that alleviating the fiscal stress of local governments has a significant effect on improving environmental efficiency. The regional green production capacity and public environmental service capacity are its transmission mechanisms. That is to say, the alleviation of fiscal stress can promote the improvement of environmental efficiency by promoting the improvement of urban green production capacity and public environmental service capacity. At the same time, the structure of fiscal funds and self-raised funds in the fixed investment of urban municipal public facilities positively adjust the urban green production capacity and public environmental service capacity, boosting the improvement effect of the policy of alleviating fiscal stress on environmental efficiency. In general, this paper has three aspects of research innovation. Firstly, based on the degree of fiscal imbalance in different cities, this paper examines the impact of fiscal stress on environmental efficiency from the horizontal dimension, which effectively complements the vertical fiscal imbalance perspective of scholars such as Lin & Zhou. Secondly, we have built a more comprehensive evaluation system for environmental governance performance. For the first time, the capacity of urban public environmental service has been scientifically quantified as public environmental services efficiency and incorporated into the assessment of regional environmental efficiency. Finally, we offer several new perspectives on heterogeneity analysis. These perspectives include the city’s economic level, administrative level, national strategic planning, and river basin factors, which provide effective theoretical and empirical support for regionally differentiated governance policies.

In the follow-up research, Section 2 focuses on the literature review and hypotheses, Section 3 introduces methodology and data, Section 4 analyzes empirical results, Section 5 discusses heterogeneity issues and mechanisms, and Section 6 summarizes the conclusions and policy implications.

## 2. Literature Review

As an indicator of ecological performance, environmental efficiency was proposed by the World Business Council for Sustainable Development (WBCSD) in 1992. In recent years, the concept of environmental efficiency has been widely used in research concerning environmental inspection and the intensity of environmental governance [12,13], the intensity of government environmental management [14], public engagement [15], environmental technological innovation [16], and fiscal environmental measures [17]. It shows that the concept of environmental efficiency is extremely valuable in many research areas.

In environmental efficiency research, the innovation of many studies is reflected in the research theme and research design. The research theme of environmental efficiency mainly includes industrial environmental efficiency and regional environmental efficiency. On the one hand, industrial environmental efficiency can also be called green total-factor productivity and environmental total factor productivity [18,19]. A large number of studies have conducted empirical research on the accounting of green production efficiency in manufacturing [20], transportation [21], and energy production [22]. On the other hand, regional environmental efficiency has also been described as green economic efficiency [23], city environmental efficiency [24], green development efficiency [25], urban environmental governance efficiency [26], eco-efficiency [27], or resource and environmental efficiency [28]. The innovation points in research design mainly come from three factors: data-driven, indicator-driven, and model-driven factors, as shown by Zhou et al., who used micro-panel data from Chinese energy companies [29]. Wu et al. introduced nitrogen dioxide as the undesirable output into the indicator system [30], and this innovation helps improve the accuracy of the measurement of the environmental efficiency of power generation companies. Wang et al. improved the traditional super-efficiency model based on the principle of material balance, which sets the threshold for pollutant emissions of sulfur dioxide, industrial soot, industrial dust, etc. When pollutant emissions exceed this threshold, the output is judged to be undesirable [31].

The theoretical origin of fiscal stress mainly comes from two fields: the western fiscal federalism theoretical system and the American public management theoretical system [32,33]. Musgrave proposed the concept of fiscal federalism in 1959 [34]. Fiscal federalism has gradually developed the first generation of fiscal federalism theory and the second generation of fiscal federalism theory [35,36]. The theory of fiscal federalism has profound explanatory power in local public goods [37]. In addition, the American public management theoretical system considers fiscal stress to be closely related to “pragmatic urbanism” or “austerity urbanism” [38,39]. Policymakers in favor of “practical municipality” advocate that local governments tend to adopt policy combinations such as income supplement, debt extension, or maintain the consistency of fiscal policy to address fiscal stress [40], while Policymakers in favor of “austerity urbanism” hold that local governments should reduce the scale of public fiscal expenditure to solve the shortage of funds for public governance [41]. It can be seen that the relationship between fiscal policy and environmental governance has a long history and a solid theoretical foundation [42].

Specific to empirical research in areas related to environmental governance, fiscal decentralization is an important explanatory variable [43,44]. Fiscal decentralization emphasizes the vertical fiscal imbalance in the fiscal system [45]. In the Chinese context, vertical fiscal imbalances lead to instability in the local government fiscal system [46]. This analytical paradigm focuses on the distribution of fiscal power between the central government and local governments [47]. However, this view ignores that local government is the provider of environmental public goods, especially as a kind of public goods. Public environmental service is also a key element of environmental governance. The supply of public goods depends more on horizontal fiscal balance, and fiscal stress is one of the key indicators of horizontal fiscal imbalances. The central fiscal budget is becoming increasingly tight, especially under the dual challenges of economic structural transformation and the COVID-19 epidemic. Horizontal fiscal balance is a more objective reflection of whether a city has the ability to withstand external risks. In order to make a good supplement to the existing literature in the field of fiscal policy, this paper studies the relationship between fiscal stress and environmental efficiency and discusses its impact mechanism. Therefore, we propose the following three research hypotheses:

**Hypothesis** **1.** *Alleviating the fiscal stress of local governments is conducive to urban environmental efficiency*.

**Hypothesis** **2.** *Green production capacity and public environmental service capacity are the intermediate mechanisms. That is, alleviating the fiscal stress promotes both the green production capacity of the city and the public environmental service capacity of the city, thereby improving the urban environmental efficiency.*

**Hypothesis** **3.** *The structure of fiscal funds and self-raised funds in the fixed investment of urban public construction positively moderates the impact of urban green production capacity and urban environmental service capacity on environmental efficiency.*

## 3. Methodology and Data

### 3.1. Model Setting

#### 3.1.1. Random-Effect Model for Testing Baseline Regression

In order to analyze the impact of fiscal stress on urban environmental efficiency, this paper establishes the following empirical model:

(1) $$EE_{it}=\beta_{0}+\beta_{1}FS_{it}+\beta Control_{it}+u_{it}$$

where the subscripts represent city i and time t, $EE_{it}$ is the explained variable, that is, urban environmental efficiency, and $FS_{it}$ is the explanatory variable, that is, the fiscal stress. $Control_{it}$ are other variables that affect urban environmental efficiency, and $u_{it}\;$ is a random error term that satisfies the independent and identical distribution.

For panel model regression, it is necessary to test which method is most suitable, such as the fixed-effect method or the random-effect method, before conducting empirical research. The key to distinguishing the models is whether unobservable individual effects are related to explanatory variables. If they are uncorrelated, choose the random-effect model; otherwise, the fixed-effect model is more appropriate. We selected the fixed-effect model and random-effect model according to the Hausman test, and the statistics of the Hausman test are as follows:

(2) $$H={\left(\hat{\beta_{RE}}-\hat{\beta_{FE}}\right)}^{\prime }{\left[Var\left(\hat{\beta_{FE}}-\hat{\beta_{RE}}\right)\right]}^{-1}{\left(\hat{\beta_{RE}}-\hat{\beta_{FE}}\right)}^{\prime }\sim \chi^{2}\left(k\right)$$

where $\hat{\beta_{RE}}$ and $\hat{\beta_{FE}}$ represent the estimated coefficients of the fixed-effect and random-effect model, respectively, and $Var\left(\hat{\beta_{FE}}-\hat{\beta_{RE}}\right)$ is the covariance matrix of the coefficient vector $\hat{\beta_{FE}}-\hat{\beta_{RE}}$. Under the null hypothesis of random-effect, H-statistic obeys the distribution of $\chi^{2}\left(k\right)$, where k is the number of explanatory variables of the regression equation. If the *p*-value of the H-statistic is greater than the critical value of 0.1, the null hypothesis cannot be rejected, then the random-effect model is accepted. Otherwise, the fixed-effect model is accepted.

The correlation of residues, including cross-sectional heteroscedasticity, temporal heteroscedasticity, and mixed heteroscedasticity, is considered in the panel model. The model is usually improved by adding robust standard errors. In addition, the feasible generalized least squares (FGLS) model is also used to estimate. Namely, the FGLS model after variable transformation satisfies the assumption of spherical disturbance term. Because the sample number (193) is much larger than the period number (6), we mainly correct the cross-sectional heteroscedasticity of the residual. Therefore, the following hypothesis is proposed:

(3) $$E\left(u_{it},\;u_{jt}|x_{i}^{*}\right)=\sigma_{i}^{2};\;E\left(\varepsilon_{it}\varepsilon_{jt}|x_{i}^{*}\right)=0$$

where $x_{i}^{*}$ includes an explanatory variable $x_{i}$ and residual $\delta_{i}$. Specifically, we use the ordinary least squares (OLS) estimation method to obtain the residual vector of the cross section and calculate the cross-sectional variance–covariance matrix. Then, using this as the weight, the weighted least squares method is used for estimation.

#### 3.1.2. Instrumental Variable Model for Dealing with the Endogeneity

Although we control more variables to minimize the endogeneity problem caused by the bias of omitted variables, improving environmental efficiency often requires the government to invest substantial fiscal resources support. When the local environmental efficiency is lower, under the stress of environmental protection, the government increases the fiscal expenditure on environmental governance, which intensifies the fiscal stress to a certain extent. The endogeneity caused by this relationship of reverse causality leads to results deviations. Endogeneity bias leads to inconsistent OLS estimates, and this inconsistency is caused by the correlation between endogenous variables and perturbation terms. The usual solution is to divide the endogenous variables into two parts. One part is related to the disturbance term, and the other part is irrelevant to the disturbance term. The part unrelated to the disturbance term is used to obtain a consistent estimate. The common method is an instrumental variable method, and the traditional instrumental variable method is the two-stage least squares method (2SLS).

In the first stage, the OLS model is used to estimate the impact of instrumental variables on endogenous variables (fiscal stress):

(4) $$FS_{it}=\mu_{0}+\mu_{1}IV_{it}+\mu Control_{it}+u_{it}$$

In the second stage, the OLS model is used to estimate the effect of the fitting values of the endogenous variables obtained in the first stage on the urban environmental efficiency:

(5) $$EE_{it}=\rho_{0}+\rho_{1}\hat{FS_{it}}+\rho Control_{it}+u_{it}$$

#### 3.1.3. Stepwise Regression Models for Testing Mediation Effects

Fiscal stress significantly affects urban green production and public environmental service levels, and the two factors are the important mechanism that affects environmental efficiency. In order to deeply explore the impact channels of fiscal stress on environmental efficiency, we use the stepwise regression method (Equations (1), (6) and (7)) to examine the mediating effect of green growth (GTFP) and public environmental service efficiency (PESE):

(6) $$M_{it}=\theta_{0}+\theta_{1}FS_{it}+\theta Control_{it}+u_{it}$$

(7) $$EE_{it}=\eta_{0}+\eta_{1}FS_{it}+\eta_{2}M_{it}+\eta Control_{it}+u_{it}$$

where $M_{it}$ is the mediator variable; they are GTFP and PESE, respectively.

#### 3.1.4. Interaction Term Regression Model for Testing Moderating Effects

The fixed-asset investment in the process of urban construction mainly comes from fiscal funds, domestic loans, funds from the bond market, foreign funds, and self-raised funds. The fiscal funds include central budget funds and local budget funds, and self-raised funds represent the hematopoietic ability of the local government’s municipal public system to ensure investment in urban public fixed assets. These two kinds of funds are currently the two most important elements to support investment in the construction of local municipal public facilities. We believe that the structure of fixed assets affects the effectiveness of the intermediary mechanism to a certain extent, which in turn affects the impact of fiscal stress on environmental efficiency. Therefore, we also include the interaction term into the model to further explore the moderating effects of fiscal funds and self-raised funds:

(8) $$EE_{it}=\gamma_{0}+\gamma_{1}FS_{it}+\gamma_{2}\Lambda_{it}+\gamma Control_{it}+u_{it}$$

where $\Lambda_{it}$ is the interaction term of the moderator variable fiscal funds (FF) and self-raised funds (SF) with the fiscal stress and mediating variables, respectively.

### 3.2. Variable Description

#### 3.2.1. Independent Variables

Fiscal stress refers to the fiscal gap formed by the continuous imbalance between the local government’s fiscal revenue and fiscal expenditure, or the mismatch between fiscal revenue and expenditure. Regarding the measurement of local fiscal stress, there are mainly three ways: the difference between fiscal expenditure and revenue, the conversion rate of tax share ratio, and the potential default rate of local debt. Theoretically, the fiscal revenue and expenditure gap is used to represent local fiscal stress. However, China’s Budget Law since 1994 has strictly prohibited local deficits and requires local budgetary revenue and expenditure to be balanced every year. The statistical fiscal gap is completely equivalent to the transfer payment from the central government to the local government. The absolute value of fiscal gap is in the accurate measuring of the government’s fiscal stress. Therefore, the relative value of the ratio between fiscal revenue (FR) and fiscal expenditure (FE) in Equation (5) is used to measure the government’s fiscal stress (FS):

(9) $$FS_{it}=FR_{it}/FE_{it}$$

where FR is the nontax revenue and the remaining tax revenue after deducting the tax allocated to the central government. FE is the payment of fiscal funds by the government to provide public goods and services and meet the common needs of the society. For the government, fiscal stress is a manifestation of the continued tension in the fiscal operation. The lower the fiscal stress, the stronger the fiscal capacity of the government is.

In addition, in order to exclude the influence of other factors on the urban environmental efficiency, we controlled for the following variables: (1) Population Growth (PG), is measured by the annual growth rate of the urban population; (2) Industrial Structure (RT), is measured by the ratio of the urban tertiary industry to GDP; (3) Residential water (RW), is an indicator used to reflect the structure of a city’s water consumption, and measured by the ratio of urban residents’ water consumption to urban total water consumption; (4) Industrial Electricity (RE), is an indicator used to reflect the structure of a city’s electricity consumption, and measured by the ratio of urban industrial water consumption to urban total water consumption; (5) Economic Growth (EG), is measured by the growth rate of urban GDP per capita; (6) Green Coverage (GC), is the ratio of the area of public green space to the built-up area in the city; (7) City Area (CA) is measured by the area of the city’s built-up area.

#### 3.2.2. Dependent Variable and Moderator Variables

Environmental efficiency was proposed by the World Business Council for Sustainable Development (WBCSD) in 1992. It is considered to be one of the most effective methods for evaluating the economy, environment, performance, and their interactions [48]. Environmental efficiency (EE) is seen as an indicator of environmental governance performance. It measures the environmental impact of per-unit economic value, which is one of the criteria for measuring sustainable development. At the same time, the estimation methods of the mediating variable green total factor productivity and public environmental service efficiency are consistent with those of the explained variable environmental efficiency. All three indicators use the Super-SBM model in DEA.

Since being pioneered by scholars such as Charles [49], DEA has rapidly grown into an exciting and fruitful field, with extensive use in management science and economics [50]. As the DEA method matures, representative efficiency measurement methods include CCR and Shannon entropy [51]. However, the mainstream model for measuring environmental efficiency is the super-efficiency model. The super-efficiency model has formed a relatively complete theoretical system [52]. In addition, the advantages of the super-efficiency model are based on relaxation measures, directional distance function measures, and curve measure evaluation methods to address undesirable outputs [53]. In terms of the exact method, the Malmquist–Luenberger index can be well adapted to the requirements of the panel data, and the decision unit can be compared in time series [54]. Therefore, the Malmquist–Luenberger index in the data envelope analysis method is used to calculate three indicators: urban environmental efficiency (EE), green total factor productivity (GTFP), and public environmental service efficiency (PESE).

Firstly, the cities in the sample can be regarded as DMUs in a Super-SBM model. Assuming that a total of m cities (DMUs) is calculated, and each DMU has n inputs (x) to produce b1 expected outputs ($y^{d}$) and b2 undesirable outputs ($y^{ud}$), the three vectors can be expressed as $x\in R^{n},y^{d}\in R^{b1},y^{ud}\in R^{b2}$. When considering m DMUs, the model can form the following three matrices:

Input:

(10) $$X=\left[x_{1},x_{2}\ldots x_{m}\right]\in R^{n*m}>0\;$$

Desirable outputs:

(11) $$Y^{d}=\left[y_{1}^{d},y_{2}^{d}\ldots y_{m}^{d}\right]\in R^{b1*m}>0\;$$

Undesirable outputs:

(12) $$Y^{ud}=\left[y_{1}^{ud},y_{2}^{ud}\ldots y_{m}^{ud}\right]\in R^{b2*m}>0\;$$

An efficiency measurement set (P) can be constructed as follows:

(13) $$P=\left\{\left(x,y^{d},y^{ud}\right)|x\rangle X\partial ,y^{d}\leq Y^{d}\partial ,y^{ud}\geq Y^{ud}\partial ,\partial \geq 0\right\}\;$$

where ∂  is the non-negative vector. Under this circumstance, to specific DMU$\left(x_{k},y_{k}^{d},y_{k}^{ud}\right),$ the formula for measuring efficiency $E^{*}\;$ in the Super-SBM model is as follows:

(14) $$E^{*}=\frac{1+\frac{1}{n}\sum_{i=1}^{n}S_{i}^{-}/x_{ik}}{1-\frac{1}{b1+b2}(\sum_{r=1}^{b1}S_{r}^{d}/y_{rk}^{d}+\sum_{k=1}^{b2}S_{k}^{ud}/y_{tk}^{ud})}$$

subject to:

(15) $$x_{ik}\geq \sum_{j=1,\neq k}^{m}x_{ij}\partial_{j}-S_{i}^{-}\;$$

(16) $$y_{rk}^{d}\leq \sum_{j=1,\neq k}^{m}y_{rj}^{d}\partial_{j}+S_{r}^{d}\;$$

(17) $$y_{tk}^{ud}\geq \sum_{j=1,\neq k}^{m}y_{tj}^{ud}\partial_{j}+S_{t}^{ud}\;$$

(18) $$1-\frac{1}{b1+b2}(\sum_{r=1}^{b1}S_{r}^{d}/y_{rk}^{d}+\sum_{k=1}^{b2}S_{k}^{ud}/y_{tk}^{ud})>0\;$$

(19) $$S^{-}\geq 0,S^{d}\geq 0,S^{ud}\geq 0,\partial \geq 0\;$$

(20) i=1,2…a;j=1,2…m(j≠k);r=1,2…b1;t=1,2…b2 

where the vector $S^{d}\;$ represents the shortage of desired outputs. The vectors $S^{-}$ and $\;S^{ud}$ are correspondingly the excesses of inputs and undesirable outputs. Environmental efficiency (*EE*) is $E^{*}$ in the above model. The value of $E^{*}$ can be greater than 1. When the value of $E^{*}$ is higher, the better environmental efficiency of a city. GTFP and PESE are also calculated the corresponding efficiency values based on the above model.”

The calculation of GTFP indicators has been supported by extensive literature. It usually puts labor capital, capital, and energy consumption in the input. Output requires considering the positive output and negative output. The positive output is mainly composed of regional GDP, and the negative output includes major pollutant emissions.

PESE  is an innovative breakthrough in the study of environmental efficiency issues. It benefits from the strong support of China’s urban construction database. Similar to the calculation method of GTFP, the input of PESE  focuses on the labor-force, fixed asset investment and construction land related to public environmental services and construction land investment in the field of public environmental services. The output of PESE mainly includes the city’s road cleaning area, sewage treatment capacity, drainage pipe network length, solid waste treatment capacity, and green space area.

Based on the EPSE and GTFP, the EE indicator further takes into account the urban disposal capacity for general industrial solid waste and water consumption. This treatment can make EE close to the actual situation of urban environmental governance. The specific data description of the three indicators are shown in Table 1.

#### 3.2.3. Instrumental Variable and Moderator Variables

Road area per capita (RAPC) is regarded as an instrumental variable in the endogenous test. In terms of the moderating effect, self-raised funds (SF) and fiscal funds (FF) are the two main funding sources of environmental infrastructure. The ratio of self-raised funds and fiscal funds in the fixed asset investment of environmental public infrastructure are used as key mechanistic variables. The variables’ details are shown in the descriptive statistics of Table 2.

### 3.3. Data Source and Application

For the variables introduced above, EE, GTFP, and PESE are efficiency indicators, and the unit is 1. We take the logarithm of the variable characterized by absolute level quantities (CA, RAPC). In addition, those variables with proportional characteristics are divided by 100 to change the unit of their value from the original% to 1 (FS, PG, RT, RW, RE, EG, FF, SF). The study sample includes 193 cities from the 29 provincial administrative divisions in China, and the sample time span ranged from 2013 to 2018. In terms of urban administrative level, these cities include 4 municipalities, 28 provincial capitals or sub-provincial cities, and 171 prefecture-level cities. At the same time, the post-text heterogeneity analysis will take into account urban agglomeration, resource-based cities, and regional environmental regulation.

The input and output data of EE, GTFP, and PESE estimation are from the “China Urban Statistical Yearbook” and “China Urban Construction Statistical Yearbook”. Self-raised funds (SF) and fiscal funds (FF) are from the “China Urban Construction Statistical Yearbook”. Fiscal revenue (FR), fiscal expenditure (FE), and other control variables are from the “China Urban Statistical Yearbook”.

## 4. Results

### 4.1. Baseline Regression

Based on the empirical model (1) in Section 3.1, we conduct an empirical analysis of the relationship between fiscal stress and environmental efficiency, and the results are shown in Table 3. Column (1) and column (2) are the random-effect model and the fixed-effect model without control variables, respectively. We find that the coefficient of fiscal stress is significantly positive at the 5% level in both models. Column (3) and column (4) are the random-effect model and the fixed-effect model with control variables added, respectively. We find that the coefficient of fiscal stress is also significantly positive in both models. The results show that alleviating fiscal stress significantly improves urban environmental efficiency, and hypothesis 1 is verified. In addition, regardless of whether the model contains control variables or not, Hausman test results show that the *p*-value is greater than 0.1. Therefore, the random-effect model is more suitable than the fixed-effect model. Considering that the cross-sectional heteroscedasticity of samples from different regions may affect the validity of the estimation results, Breusch-Pagan Test (B-P) and White test are carried out on the model. It is found that there are heteroscedasticity problems and serial correlation problems. Therefore, we re-estimate the random-effect model using the robust standard errors (OLS + Robust) in column (5) and the feasible generalized least squares method (FGLS) in column (6). The results in columns (5) and (6) show the strong stability of this relationship. The relief of fiscal stress has a significant promoting effect on local environmental efficiency. Theoretically, FGLS is more effective than OLS in large samples. Therefore, unless otherwise emphasized, the follow-up empirical studies all use the FGLS model for correlation analysis, and column (7) is used as a reference baseline for the analysis. Specifically, column (7) states that the local government’s fiscal stress is relieved by 1 unit, and the city’s environmental efficiency would increase by 0.120 units.

On the one hand, excessive fiscal stress makes the local government lack sufficient fiscal support for green subsidies and preferential policies for the environmental protection industry, resulting in a lack of enthusiasm for the research and development expenditure on green production technology, thus inhibiting environmental efficiency. On the other hand, in order to alleviate the fiscal stress, local governments are paying more attention to the construction of projects with higher and faster returns. They are more inclined to relax environmental control and introduce enterprises with excess production capacity to expand the tax base. Projects like high-tech enterprises have stricter requirements for supporting facilities, and naturally difficult to introduce. However, heavy industrial enterprises have the characteristics of a large tax base and short cycle, which can quickly increase local fiscal revenue and become the first choice of local governments, which leads to problems such as insufficient regional technological innovation, high pollution risks, and low environmental efficiency.

### 4.2. Endogenous Test

Because of the existence of heteroscedasticity, we use the Durbin–Wu–Hausman test to evaluate whether endogenous variables exist, which is more robust than the traditional Hausman test. The test result is CHI2 (1) = 64.0464 (*p* = 0.0000), indicating that the null hypothesis that “all explanatory variables are exogenous” is rejected. In order to solve the endogeneity problem, this paper uses the instrumental variable method for estimation. Whether the instrumental variable method is effective depends on the selection of instrumental variables. Instrumental variables must satisfy both correlation and exogeneity; that is, the instrumental variables are significantly correlated with explanatory variables but not with explained variables. Therefore, we applied road area per capita (RAPC) as an instrumental variable for fiscal stress. RAPC is an important factor affecting fiscal stress. The higher the road area per capita, the more perfect the city’s infrastructure construction is, that is, the city’s economic development and urbanization level will be relatively high. Furthermore, in the short term, the cost of project expenditure related to infrastructure construction will be reduced, and the government’s fiscal stress will be low. In addition, the construction of urban roads is greatly affected by the local geographical features. The exogenous nature of geographical features determines that there is little direct correlation between the development of urban roads and environmental efficiency, while urban road development affects environmental efficiency through fiscal stress. So, RAPC meets the two requirements of the instrumental variable.

Firstly, we use Hansen’s test to test whether instrumental variables are over-identified to determine the accuracy and plausibility of the results. The results of the Hansen test do not reject the null hypothesis, indicating the validity of the two instrumental variables, and there was no problem of over-identification. Secondly, we use the 2SLS estimation method to test the model, and the results are consistent with the baseline regression. However, using 2SLS may lead to “significance level distortion”, which is larger due to weak instrumental variables. So the 2SLS results are biased. Considering the sample characteristics and whether the instrumental variables are weak, the model is estimated using the limited information maximum likelihood method (LIML), which is less sensitive to weak instrumental variables. LIML is the maximum likelihood estimation based on the information of a single equation, and its estimation is valid in all estimation methods using the information of a single equation. Moreover, it has the same asymptotic distribution as 2SLS, and with better finite sample characteristics. Columns (1) and (2) show that the coefficients of LIML and 2SLS are very close, indicating that the results are robust. In addition, we confirm that there is no weak instrumental variable. 2SLS is the most efficient under the assumption of spherical perturbation terms. However, if the disturbance term has heteroscedasticity or autocorrelation, it is more effective to use the generalized estimation method of moments (GMM). At the same time, we also use the iterated GMM (IGMM) method. Because of the iterative process of IGMM, the minimum standard deviation of parameter estimation can be obtained. Therefore, to obtain a consistent estimate, we adopt 2SLS, LIML, GMM, and IGMM, four instrumental variable methods to solve the endogeneity problem. The regression results in Table 4 show that after solving endogeneity, the results remain robust, that is, less stress is associated with higher environmental efficiency.

### 4.3. Robustness Test

In the Chinese context, the relationship between fiscal stress and environmental efficiency may be disturbed by a variety of exogenous and contingent factors. They may be cyclical factors such as planning cycles and political cycles, international trade factors, administrative-level factors of cities, and variables’ measures method. Therefore, in order to make the results more robust, we consider the follow potential interference factors to retest the model, and the results are shown in Table 5.

Firstly, in terms of planning cycle factors, the planning cycle mainly refers to the national economic and social development plan, usually referred to as the five-year plan. The local government’s fiscal stress typically has significant fluctuations in the last year of the five-year planning period. The completion degree of constraint indicators in planning is the key reason for this phenomenon. Therefore, the robustness test verifies the baseline regression results after excluding the final year of the 12th Five-Year Plan (2015) data, as shown in column (1). In terms of political cycle factors, the literature on public fiscal issues all takes political cycle factors as the key explanatory variables. At present, China’s main political cycle is the National Congress of the Communist Party, and the year 2017 is the year of the 19th National Congress and a time point for the change of leading local officials. Therefore, we exclude the sample in 2017 and verify the baseline regression results, as shown in column (2). The results in columns (1) and (2) show that less fiscal stress promotes local environmental efficiency, which is consistent with the baseline regression.

Secondly, in terms of international trade factors, exogenous trade conflicts will impact the cities with export trade as the pillar industry. The fiscal stress of these cities would fluctuate because of the changes in the international trade environment. In order to avoid the impact of the conflicts of China–US trade on the result, we excluded the samples in 2018. The results are shown in column (3). We find that the coefficient of fiscal stress is very close to the baseline regression.

Then, in terms of administrative level factors, cities with high administrative levels enjoy policy care at the central fiscal fund allocation, and their fiscal stress is generally less than that of prefecture-level cities. At the same time, high administrative cities usually set stricter industrial access standards. In terms of administrative level factors, cities with high administrative levels enjoy policy care at the central fiscal fund allocation, and their fiscal stress is generally less than that of prefecture-level cities. At the same time, high administrative cities usually set stricter industrial access standards. Therefore, the robustness test excludes the samples of 4 municipalities and 15 sub-provincial planning cities, as shown in column (4). The obtained results are consistent with the baseline regression. 

Finally, excluding the interference of measurement errors of explanatory variables, we replace fiscal stress with the difference between fiscal revenue and expenditure in the model. The result is shown in column (5). Compared with the baseline regression results, the five robustness tests meet the statistical significance criteria, and the coefficient direction of fiscal stress remains unchanged. Therefore, the results are robust.

Excluding the planning cycle factors and the international trade factors, the value and significance of the regression results of the robustness test are very close to the baseline regression results. These two factors have less interference in the baseline regression conclusion. However, the regression result by excluding the political cycle factor was 0.1346, higher than the baseline regression. Moreover, the regression result excluding the interference of administrative level was 0.0734, lower than the baseline regression. It shows that the political factors have some disturbance impact on the conclusion. In other words, the impact of fiscal stress would be weakened near the end of the planning cycle. In contrast, the urban administrative level factors have a more obvious impact on the conclusion.

## 5. Discussion

### 5.1. Analysis of Heterogeneity

There are considerable differences in Chinese urban spatial location and development stage, which leads to huge differences in the fiscal expenditures of different city governments in environmental governance, such as energy conservation and emission reduction, cleaner production, and environmental infrastructure construction and operation. Therefore, we discuss the heterogeneity analysis of fiscal stress on environmental efficiency in terms of spatial location, environmental regulation, economic development, and urban characteristics.

#### 5.1.1. Spatial Location Factors

The spatial location heterogeneity of cities is divided into southern and northern cities, eastern and western cities, and central cities. The results are shown in Table 6. Column (1) is the full-sample regression result for reference. The first division method divides the samples into southern and northern cities based on China’s “Qinling-Huaihe” geographic line. The second division method divides the sample into eastern and western regions by the “Hu-Huanyong” geographic line. The reason for using geospatial location as the heterogeneity analysis is mainly to consider the spatial heterogeneity of urban development levels, industrial structures, and key objects of environmental governance in different regions. On the one hand, cities in eastern China are relatively developed areas with high population density and larger urban built-up areas, resulting in complex and diverse urban environmental problems. In comparison, cities in western China have low population density and are developing regions that produce less pollution per capita. On the other hand, traditional steel, cement, and thermal power industries are mostly distributed in cities in northern China. Therefore, air pollution is usually the key issue of environmental governance in northern cities. In contrast, phosphorus chemical, textile, printing, and dyeing industries are mostly distributed in cities in southern China, which has led to the environmental governance of southern cities focusing on water pollution. All these have resulted in significant heterogeneity in the environmental efficiency of cities in different regions of China.

Overall, regarding the results of the spatial location heterogeneity of the impact of fiscal stress on environmental efficiency, we found that the impact of fiscal stress on regional environmental efficiency has certain limitations. Specifically, for cities in southern China (column (3)), the regression results are still significant at the 1% level. The effect of fiscal stress easing on environmental efficiency has increased to 0.1572 relative to the full sample. In addition, although the significance of the regression results of the Eastern region (column (4)) is weakened, its effect is still close to that of the baseline regression (column (1)). That is to say, the improvement of environmental efficiency by fiscal policy to ease fiscal stress is still effective in the eastern region of China. However, the regression results of the northern city sample (column (2)) and the western region (column (5)) show that the promotion of regional environmental efficiency through measures to alleviate fiscal stress is not guaranteed.

#### 5.1.2. Environmental Regulation Factors

Regional pollution control and strategic environmental planning are the two most important means of environmental regulation in China, and these two policy tools have a profound impact on urban environmental efficiency. Next, we further explore the heterogeneity of the effects of fiscal policy on environmental efficiency under these two different environmental regulations. The results are shown in Table 7, and column (1) is the full-sample regression result for reference.

On the one hand, the Beijing–Tianjin–Hebei air pollution transmission channel (APTC) is the first region to implement regional pollution control. The “2017 Air Pollution Prevention and Control Work Plan in the Beijing-Tianjin-Hebei Region and its surrounding Areas” has officially designated for the first time the scope of regional environmental regulation for the joint prevention and control of air pollution. The scope covers two municipalities and 46 cities in Hebei Province, Shandong Province, Henan Province, Shanxi Province, and Inner Mongolia Autonomous Region. Column (2) shows the result of cites in the APTC area, and cities not within the APTC area were set as the control group (NAPTC), as shown in column (3). The results show that the coefficients of fiscal stress are all significantly positive at the level of at least 5%, indicating that the policy of alleviating fiscal stress has played a role in improving environmental efficiency. However, comparing the regression coefficients of the two groups of cities, we find that this fiscal policy is stronger for cities in the air pollution transmission channel than for cities outside the air pollution transmission channel.

The “Comprehensive Planning for the Yangtze River Basin 2012–2030” is the most representative water resources regulation measure for ecological protection strategic planning. It is an important basis for the development, utilization, conservation, protection of water resources, and the prevention and control of water hazards in the Yangtze River Basin. Yangtze River Basin (YRB) covers 11 provinces. Eighty-one cities in the sample belong to the YRB regions, and the regression results are shown in column (4). Cities not in the YRB area were set as the control group (NYRB), as shown in column (5). We find that the impact of the fiscal stress in the Yangtze River Basin is the highest on the urban environmental efficiency, as shown in column (4), and the positive driving effect is greater than the baseline regression, with a difference of 0.0608. This shows that the impact of the fiscal policy to alleviate the fiscal stress on promoting environmental efficiency is the most effective in the Yangtze River Basin. The fiscal stress is relieved by 1 unit, and the environmental efficiency is increased by 0.32 units. However, the results of the samples of other river basin cities have weakened in terms of significance and positive effects, indicating that to improve environmental efficiency, the effect of fiscal policy by alleviating fiscal stress is not as effective as that of cities in the Yangtze River basin. Especially for the problem of river basin environmental governance, the long-term and stable policy has a good effect on the urban environmental governance level in the Yangtze River Basin. The supervision system design of the environmental supervision system can effectively improve the environmental governance level in cities in other river basins in the short term.

#### 5.1.3. Economic Development Factors

The classical environmental Kuznets hypothesis has once expounded on the relationship between economic development factors and environmental pollution. In the process of discussing the heterogeneity caused by economic development factors, GDP and GDP per capita will be used as the main classification indicators. The study design ranked the average per capita GDP and GDP from 2013 to 2018 in 193 cities from high to low. The top 96 were composed of high-rank samples, and the bottom 97 were composed of low-rank samples. The detailed results are shown in Table 8.

Compared with the baseline regression results, in those cities with higher levels of economic development (columns (3) and (5)), fiscal stress had a stronger impact on environmental efficiency, namely 0.1513 and 0.2399, respectively. This suggests that the alleviation of fiscal stress helps improve environmental governance in cities with higher economic levels. In contrast, in those cities with lower levels of economic development (columns (2) and (4)), the results are not significant. This shows that the improvement of fiscal stress does not have a significant role in improving the level of environmental governance in these regions. Reasonably optimizing the local fiscal stress level through fiscal revenue and expenditure policies will be the top priority for improving regions with relatively high environmental governance level. The key to improving the environmental efficiency in high-level development cities lies in effectively alleviating their levels of fiscal stress. The essence of fiscal stress is to ensure a dynamic balance between fiscal revenue and fiscal expenditure. In terms of fiscal revenue, a diversified and green local tax base helps to ensure the stability of the fiscal revenue system. At the same time, regional environmental policy objectives should take into account the stability of the tax base and focus on implementing a long-term and sustainable plan rather than short-term administrative intervention or fines. In terms of fiscal expenditure, local governments in areas with high development levels should alleviate the fiscal stress caused by urbanization and effectively control the rapid influx process. In other words, the pace of urbanization should match the growth rate of the scale of urban fiscal expenditure. From the objective law of urban development, high-speed urbanization and population agglomeration will produce a large number of the public environmental service needs, such as garbage disposal and sewage disposal. These public environmental service needs are bound to expand the scale of urban fiscal expenditure.

#### 5.1.4. Urban Characteristic Factors

We believe that urban resource endowment characteristics affect the heterogeneity of fiscal policy implementation. In 2013, the central government released the “National Sustainable Development Plan for Resource-based Cities (2013–2020)”, which includes 73 resource-based cities. These resource-based cities are highly dependent on the stock of ore resources. The fiscal revenue of resource-based cities is often affected by the price of resources in the secondary market. At the same time, the industrial structure of such cities is relatively single, and most of them are pollution-intensive industries. Therefore, whether the environmental efficiency of such cities is sensitive to fiscal stress requires special attention. As the results in Table 9, the results in columns (2) and (3) show that the impact of resource-based city fiscal stress on environmental efficiency is positive but not significant. In contrast, the fiscal stress of non-resource-based cities is significantly positive for environmental efficiency at the 1% level. It is 0.0202 percentage points higher than the baseline regression results, as shown in column (3). The fiscal situation of resource-based cities is too dependent on the prosperity of industries related to resource development and utilization and is vulnerable to the impact of resource and mineral prices and external environmental protection policies. In the short term, fiscal policy tools do not have a significant regulatory role in the environmental efficiency of resource-based cities. However, the environmental efficiency of non-resource-based cities is more sensitive to fiscal stress because of their diversified fiscal revenue structure. This has led to a long-term stable FS level in non-resource-based cities. In the long run, a stable level of fiscal stress can better promote the efficiency and sustainability of environmental governance in non-resource cities. This is undoubtedly beneficial to its environmental efficiency.

On the other hand, an urban agglomeration is also an important factor affecting the heterogeneity of fiscal policy implementation. Due to the influence of the functional division of the urban agglomeration and the spatial layout of the industrial chain, the urban environmental efficiency within the urban agglomeration is more sensitive to fiscal stress. In the process of regional integration, the environmental regulation policies in urban agglomeration show synergy and consistency, which is the main reason for considering the urban agglomeration factors in the heterogeneity analysis. Therefore, the Beijing-Tianjin-Hebei urban agglomeration, the Yangtze River Delta urban agglomeration, and the Guangdong-Hong Kong-Macao urban agglomeration have become the main research objects, the results are shown in columns (4)–(5) of Table 9. Column (4) are mainly the cites composition of the above three urban agglomerations. Column (5) presents the results of the samples outside of the urban agglomeration. It can clearly reflect the heterogeneity problem caused by urban agglomeration factors. Column (4) showed that fiscal stress mitigation in the urban agglomeration structure has a greater positive effect on the environmental efficiency, reaching 0.1553, while the result in column (5) is not significant. This is consistent with our previous analysis and the actual situation.

### 5.2. Mediating Effect and Moderating Effect

#### 5.2.1. Mediating Effect

Improving environmental efficiency mainly includes two paths: improving the green production level and the public environmental service capacity. The green total factor productivity (GTFP) represents the green clean production level of the urban production activity, and public environmental service efficiency (PESE) represents urban government public environmental service capacity. We use stepwise regression models (1)–(3) to test the mediating effect of GTFP and PESE on environmental efficiency improvement in the process of fiscal policy implementation, and the results are shown in Table 10.

Specifically, in column (2), the easing of fiscal stress has a significantly positive coefficient on green total factor productivity at the 1% level. At the same time, the coefficient of GTFP in column (3) is also significantly positive at the 1% level, and the coefficient of FS is not significant. Therefore, it is shown that local green total factor productivity plays a fully mediating role in the impact of fiscal stress on environmental efficiency. That is to say, the alleviation of the urban fiscal stress affects the overall urban environmental efficiency by improving the urban green production level.

Similarly, for path 2 public environmental service capacity, the coefficient of fiscal stress mitigation on public service capacity (column (4)) is significantly positive at the 5% level. At the same time, the coefficient of EPCE in column (5) is also significantly positive at the 1% level, and the coefficient of fiscal stress is significantly positive at the 1% level. However, compared with the baseline regression in column (1), the size of fiscal stress is weakened, and it is shown that local public environmental service capacity plays a partial mediating effect of fiscal stress on environmental efficiency. That is to say, the alleviation of the urban fiscal stress affects the overall environmental efficiency of the city by improving the urban public environmental service capacity.

By comparing the two mediating effects, the mediating effect of GTFP is higher than that of PESE. Therefore, this result can indirectly explain why local governments are willing to invest more environmental governance resources to improve the level of green production.

#### 5.2.2. Moderating Effect

The above analysis found that green production and public environmental service capacity are the intermediate channels through which fiscal stress affects environmental efficiency. However, we believe that there is an important moderating variable that can make the above two pathways work better. Due to the characteristics of the source and usage of fixed assets, we believe that the fiscal funds and self-raised funds in fixed assets may be this black box. The proportion of fiscal funds and self-raised funds in fixed investment can be regarded as a leverage tool to a certain extent. Moreover, if this leverage tool can be tested, it will help propose more precise policy control measures. Therefore, we use model (8) to re-regress the three interaction terms, and the results are shown in Table 11. Column (1) is the regression result covering the triple interaction term of fiscal stress, green total factor productivity, and fiscal funding. Column (2) is the regression result covering the triple interaction term of fiscal stress, public environmental service efficiency, and fiscal funding. Column (3) is the regression result covering the triple interaction term of fiscal stress, green total factor productivity and self-raised funds, and column (4) is the regression result covering the triple interaction term of fiscal stress, public environmental service efficiency and self-raised funds.

By comparing the coefficients of the interaction terms in columns (1) and (2), we found that only the coefficients of the interaction terms in column (1) are significantly positive at the 1% level, but column (2) is not significant. It shows that the proportion of fiscal funds in fixed assets only moderates the mediating effect of green total factor productivity. However, the results of the interaction terms in columns (3) and (4) are significantly positive, at least at the 1% level, The significance and magnitude of the coefficients of the interaction terms in column (4) are greater than those in column (3). It means that the proportion of self-raised funds in fixed assets has a significant moderating effect on both green production and public environmental service capabilities and has an obvious and strong leverage effect on the mechanism of public environmental service capabilities. The funding structure of environmental fixed asset can become a multiplier for alleviating fiscal stress, improving local public environmental service capacity, and improving green production capacity. In particular, in some cities, the government’s fixed-asset investment in public infrastructure is mainly dependent on self-raised funds. Fiscal policies to ease fiscal stress are more important. This shows that the fixed asset investment system is an important bridge between fiscal and environmental governance system. It is worth emphasizing that funding structure of environmental infrastructure reflects urban policy sustainability in the process of environmental governance.

## 6. Conclusions

Overall, fiscal stress has a significant impact on environmental efficiency. Specifically, the less fiscal stress, the more environmental efficient it is. In other words, fiscal balance contributes to improving urban environmental governance performance. This conclusion remains robust, excluding interferences such as political cycles, planning cycles, trade frictions, and administrative hierarchies. However, the results of the heterogeneity analysis show that the environmental governance of Chinese cities is highly different and complex. In the south, in the Yangtze River basin and urban agglomerations area, alleviating fiscal stress can improve the environmental efficiency of cities. However, environmental efficiency in areas with lagging levels of development is not sensitive to fiscal stress, and similar conclusions are also found in resource-based cities. In terms of the mediation effect, green production and public environmental services are effective policy paths. Fiscal stress can affect environmental efficiency through these two paths. In terms of the regulatory role, the funding structure of environmental governance projects is an important policy regulation fulcrum. Local governments should maintain a balance between fiscal funds and self-raised funds. A reasonable capital structure is an important lever to regulate environmental efficiency.

On the basis of the above conclusions, we put forward policy suggestions from four aspects: fiscal revenue, fiscal expenditure, policy regulation and control, and capital structure. In terms of fiscal revenue, local governments should maintain the stability of the tax base. Environmental regulatory policies must not destabilize the tax base. At the same time, collecting a special environmental governance tax is helpful in supplementing the city’s fiscal revenue and ensuring the stability of environmental governance funds. In terms of fiscal expenditure, local governments should ensure technological innovation in the green production of industrial enterprises through continuous fiscal expenditure. At the same time, fiscal expenditure on public environmental services should maintain steady growth year by year. In terms of policy regulation, local governments should adopt more accurate environmental policy tools. Urban characteristics such as spatial location, development level, strategic positioning, and industrial structure should be fully valued. In terms of funding structure, environmental governance funds should not rely too much on the fiscal budget. Local governments should enhance the multichannel fundraising of environmental governance projects and optimize the structure of environmental governance funds.

## Figures and Tables

**Table 1:** Inputs and outputs on the evaluation of EE, GTFP, and PESE indicators.

Item	Specific Data Name	GTFP	PESE	EE
Input	Labor force employment	√	−	√
	Capital Stock	√	−	−
	Fixed Assets Investment	−	−	√
	Total electricity consumption	√	−	√
	Total water consumption	−	−	√
	Public environmental service practitioners	−	√	−
	Fixed Assets Investment in public environmental services ^a^	−	√	−
	Public environmental service construction land ^b^	−	√	−
Desirable output	GDP	√	−	√
	Utilization rate of general industrial solid waste	−	−	√
	Road clearance area	−	√	√
	Total length of drainage pipe	−	√	√
	Total sewage treatment	−	√	√
	Amount of dry sludge disposal	−	√	√
	Harmless disposal amount of household garbage	−	√	√
	Green area of park	−	√	√
Undesirable output	Discharge of industrial wastewater	√	−	√
	Industrial sulfur dioxide emissions	√	−	√
	Industrial soot emission	√	−	√

Note: ^a^ fixed assets investment in public environmental services are the sum of fixed asset investments related to environmental governance; ^b^ public environmental service construction land is the sum of public land related to environmental governance.

**Table 2:** Descriptive Statistics for Variables.

Variables	N	Mean	Sd.	Min	Max
EE	1158	0.8145	0.3177	0.0008	2.9989
FS	1158	0.5346	0.2248	0.1152	1.5968
PG	1158	2.3436	1.4383	−3.2189	7.1405
RT	1158	0.4264	0.0993	0.1644	0.8098
RW	1158	0.4118	0.1348	0.0514	0.9437
RE	1158	0.6618	0.1563	0.1190	0.9724
EG	1158	0.0682	0.1369	−0.9999	1.5473
CA	1158	9.2328	0.8106	7.0909	11.4078
GC	1158	0.4094	0.0415	0.1476	0.5811
RAPC	1158	1.6711	0.5534	0.4291	4.6356
GTFP	1158	0.4277	0.2464	0.0557	1.9966
PESE	1158	0.2519	0.3947	0.0051	9.5701
FF	1158	0.3218	0.3059	0.0000	1.0000
SF	1158	0.3150	0.2904	0.0000	1.0000

**Table 3:** The baseline regression on the impact of fiscal stress on environmental efficiency.

Variables	(1)	(2)	(3)	(4)	(5)	(6)
	RE	FE	RE	FE	RE	FGLS
	EE	EE	EE	EE	EE	EE
FS	0.1481 **	0.2128 **	0.1290 **	0.0386 *	0.1290 **	0.1204 ***
	(0.0611)	(0.0965)	(0.0628)	(0.0987)	(0.0604)	(0.0300)
PG			−0.0420 ***	−0.0084 *	−0.0420 ***	−0.0448 ***
			(0.0061)	(0.0119)	(0.0074)	(0.0046)
RT			−0.3207 ***	−0.1812 *	−0.3207 ***	0.0339
			(0.1128)	(0.2024)	(0.1219)	(0.0723)
RW			0.2873 ***	0.3632 ***	0.2873 ***	0.3212 ***
			(0.0829)	(0.0976)	(0.0982)	(0.0554)
RE			−0.4401 ***	−0.2718 **	−0.4401 ***	−0.5248 ***
			(0.0805)	(0.1071)	(0.0958)	(0.0439)
EG			0.1234 **	0.0671	0.1234 *	0.1363 **
			(0.0518)	(0.0506)	(0.0849)	(0.0633)
GC			−0.2989	−0.1218	−0.2989	−0.6442 ***
			(0.2615)	(0.3054)	(0.3672)	(0.1884)
CA			−0.0339 *	0.0868 *	−0.0339 *	−0.0480 ***
			(0.0196)	(0.0832)	(0.0253)	(0.0086)
_cons	0.7353 ***	0.7008 ***	1.5810 ***	0.3269 *	1.5810 ***	1.7726 ***
	(0.0368)	(0.0520)	(0.2410)	(0.7933)	(0.3104)	(0.1298)
Hausman *p*-value	0.3864		0.6451			
N	1158	1158	1158	1158	1158	1158

Standard errors in parentheses, * *p* < 0.10, ** *p* < 0.05, *** *p* < 0.01.

**Table 4:** The empirical results on endogeneity tests for models.

Variables	(1)	(2)	(3)	(4)
	2SLS	LIML	GMM	IGMM
	EE	EE	EE	EE
FS	0.3358 ***	0.3454 ***	0.3405 ***	0.3397 ***
	(0.0886)	(0.0909)	(0.0890)	(0.0890)
PG	−0.0512 ***	−0.0511 ***	−0.0487 ***	−0.0484 ***
	(0.0079)	(0.0079)	(0.0079)	(0.0079)
RT	−0.1268	−0.1341	−0.1306	−0.1271
	(0.1199)	(0.1212)	(0.1203)	(0.1203)
RW	0.3842 ***	0.3875 ***	0.3684 ***	0.3645 ***
	(0.0820)	(0.0823)	(0.0821)	(0.0821)
RE	−0.4856 ***	−0.4861 ***	−0.4940 ***	−0.4963 ***
	(0.0697)	(0.0698)	(0.0701)	(0.0701)
EG	0.1510	0.1509	0.0678	0.0555
	(0.0977)	(0.0977)	(0.0979)	(0.0986)
GC	−0.6682 **	−0.6784 **	−0.6638 **	−0.6508 **
	(0.2698)	(0.2707)	(0.2706)	(0.2706)
CA	−0.0203	−0.0196	−0.0161	−0.0164
	(0.0149)	(0.0150)	(0.0149)	(0.0149)
_cons	1.4233 ***	1.4172 ***	1.3889 ***	1.3882 ***
	(0.2082)	(0.2089)	(0.2080)	(0.2079)
N	1158	1158	1158	1158

Standard errors in parentheses, ** *p* < 0.05, *** *p* < 0.01.

**Table 5:** The empirical results on robustness tests for models.

Variables	(1)	(2)	(3)	(4)	(5)
	Planning Cycle	Political Cycle	International Trade	Administrative Level	Measured Deviation
	EE	EE	EE	EE	EE
FS	0.1222 ***	0.1346 ***	0.1225 ***	0.0734 *	0.1163 ***
	(0.0328)	(0.0370)	(0.0247)	(0.0403)	(0.0042)
PG	−0.0450 ***	−0.0449 ***	−0.0483 ***	−0.0577 ***	−0.0500 ***
	(0.0049)	(0.0050)	(0.0055)	(0.0052)	(0.0046)
RT	−0.0017	−0.0201	−0.0753	−0.1938**	0.0696
	(0.0807)	(0.0827)	(0.0592)	(0.0863)	(0.0654)
RW	0.3164 ***	0.3297 ***	0.2751 ***	0.3819 ***	0.3176 ***
	(0.0604)	(0.0591)	(0.0456)	(0.0595)	(0.0537)
RE	−0.5003 ***	−0.4942 ***	−0.5235 ***	−0.5360 ***	−0.5076 ***
	(0.0494)	(0.0455)	(0.0341)	(0.0489)	(0.0420)
EG	0.1637 **	0.1481 **	0.1273 **	0.1131 *	0.1340 **
	(0.0702)	(0.0725)	(0.0579)	(0.0646)	(0.0625)
GC	−0.7881 ***	−0.5660 ***	−0.6504 ***	−0.4577 **	−0.5094 ***
	(0.2082)	(0.1927)	(0.1664)	(0.2086)	(0.1846)
CA	−0.0576 ***	−0.0362 ***	−0.0417 ***	−0.0674 ***	−0.0610 ***
	(0.0096)	(0.0091)	(0.0081)	(0.0094)	(0.0091)
_cons	1.9323 ***	1.6226 ***	1.7964 ***	1.9939 ***	1.8644 ***
	(0.1439)	(0.1339)	(0.1141)	(0.1387)	(0.1294)
N	965	965	965	1056	1158

Standard errors in parentheses, * *p* < 0.10, ** *p* < 0.05, *** *p* < 0.01.

**Table 6:** Empirical analysis of spatial location heterogeneity factors.

Variables	(1)	(2)	(3)	(4)	(5)
	Baseline	North Cities	South Cities	Eastern Region	Western Region
	EE	EE	EE	EE	EE
FS	0.1204 ***	0.0287	0.1572 ***	0.1163 **	0.0802
	(0.0300)	(0.0392)	(0.0434)	(0.0583)	(0.0580)
PG	−0.0448 ***	−0.0261 ***	−0.0634 ***	−0.0477 ***	−0.0147 *
	(0.0046)	(0.0042)	(0.0062)	(0.0058)	(0.0091)
RT	0.0339	−0.1764 **	0.2225 **	0.1166	−0.3439 **
	(0.0723)	(0.0850)	(0.1015)	(0.1158)	(0.1462)
RW	0.3212 ***	0.3900 ***	0.3708 ***	0.2561 ***	0.4894 ***
	(0.0554)	(0.0492)	(0.0861)	(0.0778)	(0.1028)
RE	−0.5248 ***	−0.3101 ***	−0.7125 ***	−0.6731 ***	−0.2959 ***
	(0.0439)	(0.0584)	(0.0631)	(0.0734)	(0.0752)
EG	0.1363 **	0.1316 ***	0.1179 *	0.0825 *	0.0700 *
	(0.0633)	(0.0440)	(0.1055)	(0.0506)	(0.0730)
GC	−0.6442 ***	−0.2295	−0.5291 **	0.0633 *	0.1155 **
	(0.1884)	(0.1890)	(0.2279)	(0.2213)	(0.5294)
CA	−0.0480 ***	−0.0573 ***	−0.0508 ***	−0.0741 ***	−0.0798 ***
	(0.0086)	(0.0151)	(0.0116)	(0.0156)	(0.0166)
Constant	1.7726 ***	1.6862 ***	1.7136 ***	1.7870 ***	1.7267 ***
	(0.1298)	(0.1794)	(0.1720)	(0.1934)	(0.2851)
N	1158	498	660	882	276

Standard errors in parentheses, * *p* < 0.10, ** *p* < 0.05, *** *p* < 0.01.

**Table 7:** Empirical analysis of environmental regulation heterogeneity factors.

Variables	(1)	(2)	(3)	(4)	(5)
	Baseline	APTC	NAPTC	YRB	NYRB
	EE	EE	EE	EE	EE
FS	0.1204 ***	0.1803 **	0.1083 ***	0.1812 ***	0.0967 **
	(0.0300)	(0.0843)	(0.0364)	(0.0457)	(0.0420)
PG	−0.0448 ***	−0.0460 ***	−0.0571 ***	−0.0525 ***	−0.0478 ***
	(0.0046)	(0.0082)	(0.0053)	(0.0064)	(0.0069)
RT	0.0339	−0.0996	0.4501 ***	0.1748	−0.1229
	(0.0723)	(0.1441)	(0.0880)	(0.1228)	(0.0940)
RW	0.3212 ***	0.5604 ***	0.5692 ***	0.3420 ***	0.4313 ***
	(0.0554)	(0.0919)	(0.0675)	(0.0966)	(0.0671)
RE	−0.5248 ***	−0.5359 ***	−0.3352 ***	−0.8461 ***	−0.3990 ***
	(0.0439)	(0.1116)	(0.0486)	(0.0723)	(0.0550)
EG	0.1363 **	0.0460	0.1228	0.1632	0.1603 **
	(0.0633)	(0.0546)	(0.0811)	(0.1263)	(0.0659)
GC	−0.6442 ***	0.5980	0.3885 **	0.0054	−0.6928 ***
	(0.1884)	(0.3768)	(0.1767)	(0.2701)	(0.2590)
CA	−0.0480 ***	−0.0856 ***	0.0548 ***	−0.0552 ***	−0.0748 ***
	(0.0086)	(0.0187)	(0.0077)	(0.0127)	(0.0114)
Constant	1.7726 ***	1.6680 ***	1.2170 ***	1.6293 ***	2.0433 ***
	(0.1298)	(0.2842)	(0.1842)	(0.1899)	(0.1749)
N	1158	294	864	486	672

Standard errors in parentheses, ** *p* < 0.05, *** *p* < 0.01.

**Table 8:** Empirical analysis of economic development heterogeneity factors.

Variables	(1)	(2)	(3)	(4)	(5)
		Per_GDP		GDP	
	Baseline	Low Rank	High Rank	Low Rank	High Rank
	EE	EE	EE	EE	EE
FS	0.1204 ***	0.0730	0.1513 ***	0.5161	0.1312 *
	(0.0300)	(1.2753)	(0.0467)	(1.3965)	(0.0676)
PG	−0.0448 ***	−0.1106 ***	−0.0349 ***	−0.0819	−0.0496 ***
	(0.0046)	(0.0388)	(0.0053)	(0.0553)	(0.0086)
RT	0.0339	−0.7124	0.3193 ***	−1.4834	0.1145
	(0.0723)	(0.7559)	(0.0805)	(1.3897)	(0.1439)
RW	0.3212 ***	1.2468	0.2378 ***	0.6144	0.3429 ***
	(0.0554)	(0.9636)	(0.0835)	(0.5032)	(0.1114)
RE	−0.5248 ***	−1.2396 *	−0.4669 ***	−1.3706 *	−0.4229 ***
	(0.0439)	(0.6635)	(0.0613)	(0.7484)	(0.0954)
EG	0.1363 **	0.1343	0.0461	−0.0644	0.1084
	(0.0633)	(0.1426)	(0.0788)	(0.2483)	(0.0994)
GC	−0.6442 ***	−2.2307	−0.0153	−0.8478	0.2937
	(0.1884)	(1.7913)	(0.2454)	(2.7893)	(0.3098)
CA	−0.0480 ***	0.4766	−0.0004	0.1291	−0.0207
	(0.0086)	(0.3154)	(0.0107)	(0.1986)	(0.0168)
Constant	1.7726 ***	−2.4801	0.9166 ***	1.3547	1.0154 ***
	(0.1298)	(3.3391)	(0.1590)	(1.8728)	(0.2551)
N	1158	582	576	582	576

Standard errors in parentheses, * *p* < 0.10, ** *p* < 0.05, *** *p* < 0.01.

**Table 9:** Empirical analysis of urban characteristic heterogeneity factors.

Variables	(1)	(2)	(3)	(4)	(5)
	Baseline	Resource-Based Cities	Non-Resource Cities	Urban Agglomerations	Non-Urban Agglomerations
	EE	EE	EE	EE	EE
FS	0.1204 ***	0.0155	0.1406 ***	0.1553 ***	0.0961
	(0.0300)	(0.0978)	(0.0337)	(0.0479)	(0.0658)
PG	−0.0448 ***	−0.0609 ***	−0.0383 ***	−0.0246 ***	−0.0638 ***
	(0.0046)	(0.0115)	(0.0051)	(0.0059)	(0.0090)
RT	0.0339	−0.4096 **	0.2271 ***	0.0842	−0.0737
	(0.0723)	(0.1894)	(0.0851)	(0.0913)	(0.1340)
RW	0.3212 ***	0.5201 ***	0.1733 **	0.3568 ***	0.3213 ***
	(0.0554)	(0.1221)	(0.0761)	(0.0897)	(0.0939)
RE	−0.5248 ***	−0.4055 ***	−0.4933 ***	−0.7865 ***	−0.3856 ***
	(0.0439)	(0.1180)	(0.0606)	(0.0754)	(0.0766)
EG	0.1363 **	0.2241 **	0.1343 *	0.0627	0.1713 **
	(0.0633)	(0.1025)	(0.0775)	(0.0894)	(0.0817)
GC	−0.6442 ***	−0.1993	−0.8038 ***	−0.4809 *	−0.5842 **
	(0.1884)	(0.4072)	(0.2239)	(0.2511)	(0.2879)
CA	−0.0480 ***	−0.0194	−0.0322 ***	−0.0376 ***	−0.0381 **
	(0.0086)	(0.0181)	(0.0106)	(0.0111)	(0.0153)
_cons	1.7726 ***	1.4002 ***	1.6195 ***	1.6856 ***	1.6437 ***
	(0.1298)	(0.2879)	(0.1589)	(0.1601)	(0.2255)
N	1158	438	720	420	738

Standard errors in parentheses, * *p* < 0.10, ** *p* < 0.05, *** *p* < 0.01.

**Table 10:** Analysis of mediating effect—Green Production and Public Environmental Service.

Variables	(1)	(2)	(3)	(4)	(5)
	Baseline	Paths I		Paths II	
	EE	GTFP	EE	PESE	EE
FS	0.1204 ***	0.3081 ***	0.0232	0.1075 ***	0.0820 ***
	(0.0300)	(0.0203)	(0.0344)	(0.0168)	(0.0257)
GTFP			0.4054 ***		
			(0.0249)		
PESE					0.1107 ***
					(0.0120)
PG	−0.0448 ***	0.0016	−0.0531 ***	−0.0043 *	−0.0346 ***
	(0.0046)	(0.0024)	(0.0046)	(0.0024)	(0.0026)
RT	0.0339	0.0212	0.1162	0.1720 ***	0.0236
	(0.0723)	(0.0385)	(0.0729)	(0.0390)	(0.0456)
RW	0.3212 ***	0.0106	0.5683 ***	−0.0300	0.3067 ***
	(0.0554)	(0.0240)	(0.0564)	(0.0222)	(0.0324)
RE	−0.5248 ***	−0.3677 ***	−0.1071 **	0.0489 **	−0.3736 ***
	(0.0439)	(0.0225)	(0.0439)	(0.0205)	(0.0326)
EG	0.1363 **	0.1241 ***	0.0487	0.0187	0.0841 ***
	(0.0633)	(0.0222)	(0.0555)	(0.0261)	(0.0292)
GC	−0.6442 ***	−0.1900 ***	0.5709 ***	0.1535 *	−0.3394 **
	(0.1884)	(0.0705)	(0.1435)	(0.0898)	(0.1497)
CA	−0.0480 ***	0.0010	0.0339 ***	−0.0454 ***	−0.0525 ***
	(0.0086)	(0.0037)	(0.0064)	(0.0048)	(0.0114)
_cons	1.7726 ***	0.5096 ***	0.9841 ***	0.3957 ***	1.5709 ***
	(0.1298)	(0.0529)	(0.1366)	(0.0650)	(0.1311)
N	1158	1158	1158	1158	1158

Standard errors in parentheses, * *p* < 0.10, ** *p* < 0.05, *** *p* < 0.01.

**Table 11:** Analysis of moderating effect—Fixed Assets Investment Financing System.

Variables	(1)	(2)	(3)	(4)
	EE	EE	EE	EE
FS	0.1013 ***	0.0939 ***	0.0637 *	0.0755 **
	(0.0246)	(0.0313)	(0.0330)	(0.0308)
M1 (FS * GTFP * FF)	0.1551 ***			
	(0.0307)			
M2 (FS * PESE * FF)		0.3600		
		(0.0801)		
M3 (FS * GTFP * SF)			0.3118 ***	
			(0.0662)	
M4 (FS * PESE * SF)				0.4689 ***
				(0.0570)
PG	−0.0356 ***	−0.0457 ***	−0.0453 ***	−0.0440 ***
	(0.0026)	(0.0046)	(0.0044)	(0.0046)
RT	−0.0001	−0.0070	0.0020	−0.0103
	(0.0475)	(0.0752)	(0.0747)	(0.0741)
RW	0.3280 ***	0.3401 ***	0.3226 ***	0.3333 ***
	(0.0337)	(0.0557)	(0.0555)	(0.0542)
RE	−0.3941 ***	−0.5427 ***	−0.5512 ***	−0.5708 ***
	(0.0318)	(0.0447)	(0.0450)	(0.0441)
EG	0.0971 ***	0.1367 **	0.1206 *	0.1278 **
	(0.0317)	(0.0635)	(0.0616)	(0.0636)
GC	−0.3209 **	−0.6028 ***	−0.5719 ***	−0.6301 ***
	(0.1524)	(0.1895)	(0.1873)	(0.1854)
CA	−0.0516 ***	−0.0454 ***	−0.0486 ***	−0.0436 ***
	(0.0094)	(0.0088)	(0.0084)	(0.0086)
_cons	1.5938 ***	1.7545 ***	1.7878 ***	1.7716 ***
	(0.1193)	(0.1289)	(0.1269)	(0.1296)
N	1158	1158	1158	1158

Standard errors in parentheses, * *p* < 0.10, ** *p* < 0.05, *** *p* < 0.01.

## Data Availability

The data used to support the findings of this study are available from the corresponding author upon request.
